# Supplementary material for: GAF-dependent chromatin plasticity determines promoter usage to mediate locust gregarious behavior
Source: EMBO J. 2025 Apr 7;44(10):2928–48. doi: 10.1038/s44318-025-00428-x (PMC12084303; doi:10.1038/s44318-025-00428-x)
Supplement: Supplementary file 1 — Appendix [file 44318_2025_428_MOESM1_ESM.pdf]

Appendix

GAF-dependent Chromatin Plasticity Determines Promoter Usage to Mediate Locust Gregarious Behavior

Xiao Li[1, 2, *], Feng Jiang[1, 2, *], Qing Liu[1], Zizheng Zhang[1, 2], Wenjing Fang[1, 2], Yutong Wang[1], Hongran Liu[1, 2], Le Kang[1, 2, 3, #]

[1] State Key Laboratory of Integrated Management of Pest Insects and Rodents, Institute of Zoology, Chinese Academy of Sciences, Beijing, China

[2] University of Chinese Academy of Sciences, Beijing, China

[3] College of Life Science, Hebei University, Baoding, China

*These authors contributed equally to this study.

#Corresponding author:

Le Kang, Ph.D

CAS Distinguished Professor

Institute of Zoology, Chinese Academy of Sciences (CAS)

Beijing 100101, China

Tel: 86-10-6480-7219

Fax: 86-10-6480-7099

E-mail: lkang@ioz.ac.cn

Table of Contents

Appendix Figure S1	Pages 3
Appendix Figure S2	Pages 4
Appendix Figure S3	Pages 5
Appendix Figure S4	Pages 6
Appendix Figure S5	Pages 7
Appendix Figure S6	Pages 8
Appendix Figure S7	Pages 9
Appendix Figure S8	Pages 10
Appendix Figure S9	Pages 11
Appendix Table S1	Pages 12-13
Appendix Table S2	Pages 13-14

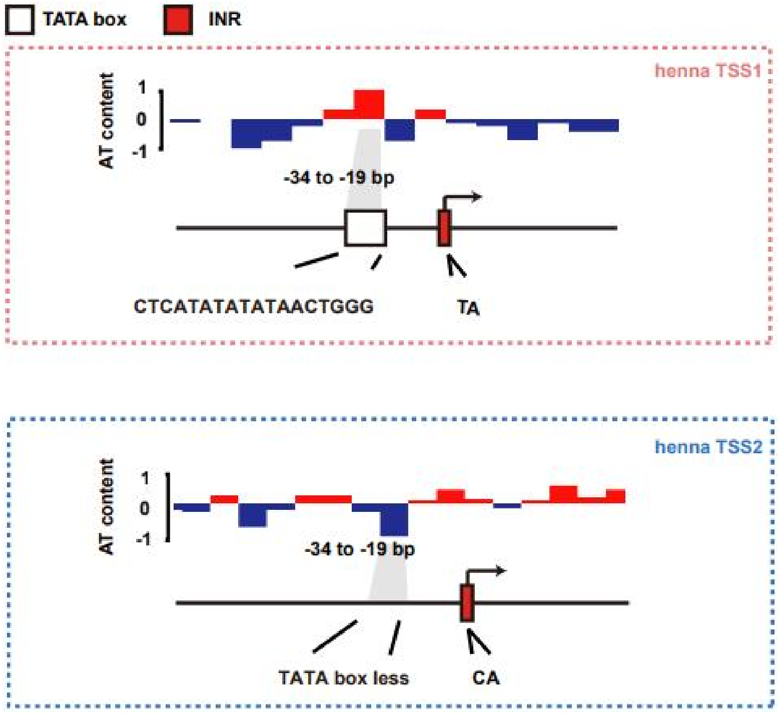

Appendix Figure S1. Different compositions of core promoter motifs at *henna* TSS1 and TSS2. For TSS1, it contains a TATA box at -34 to -19 bp and an initiator (INR). For TSS2, it contains an INR.

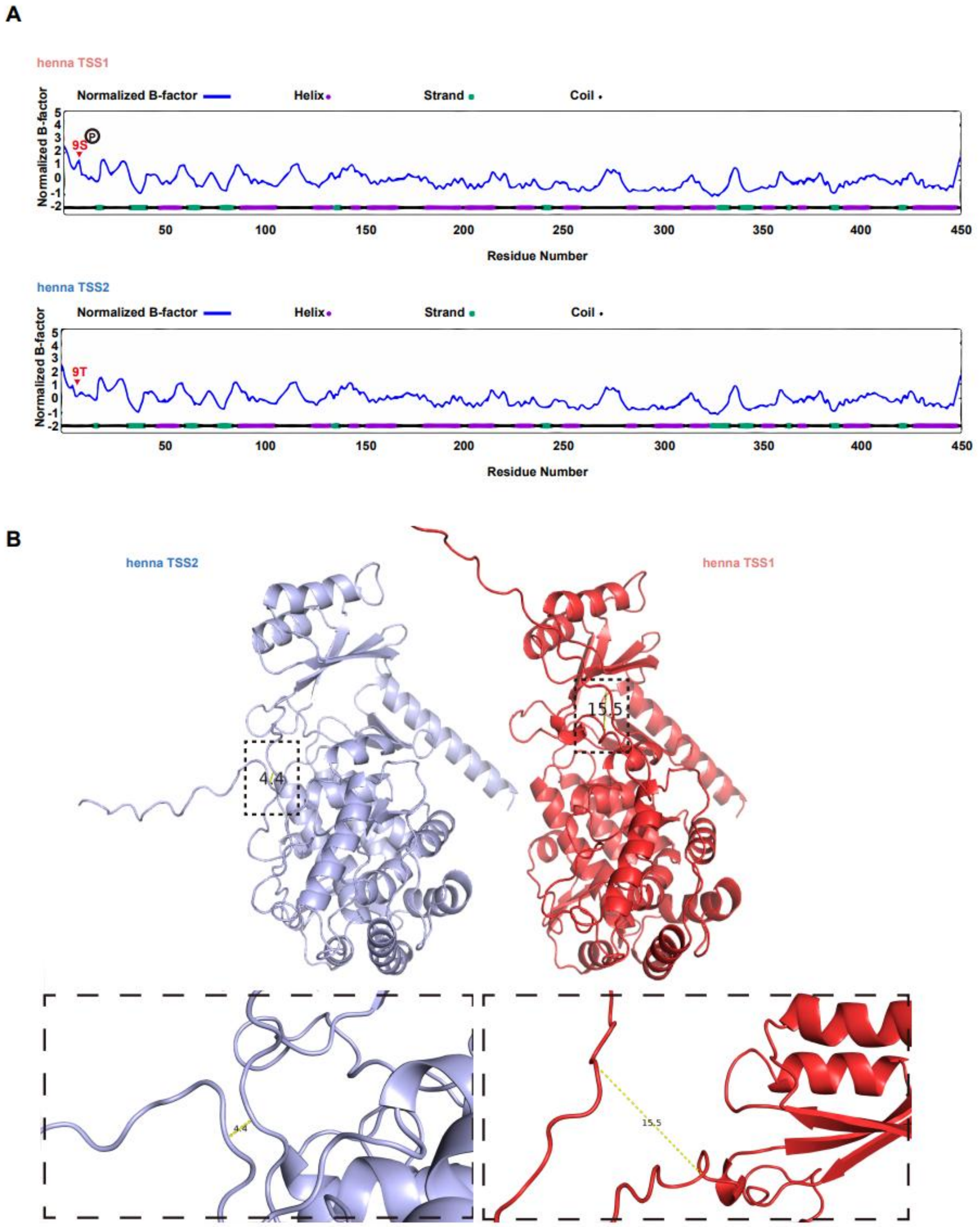

Appendix Figure S2. Predicted protein structures of TSS1 and TSS2 by I-TASSER and AlphaFold.

A The secondary structure was predicted by I-TASSER. A phosphorylated site Ser^9 was predicted for TSS1, but this site was absent for TSS2.

B Three-dimensional structures were predicted by AlphaFold, displaying as relaxed models. Red color represents the structure of TSS1, and light blue color represents the structure of TSS2. Black dotted boxes display the intra distance between the sixteenth and the twenty-nineth amino acids.

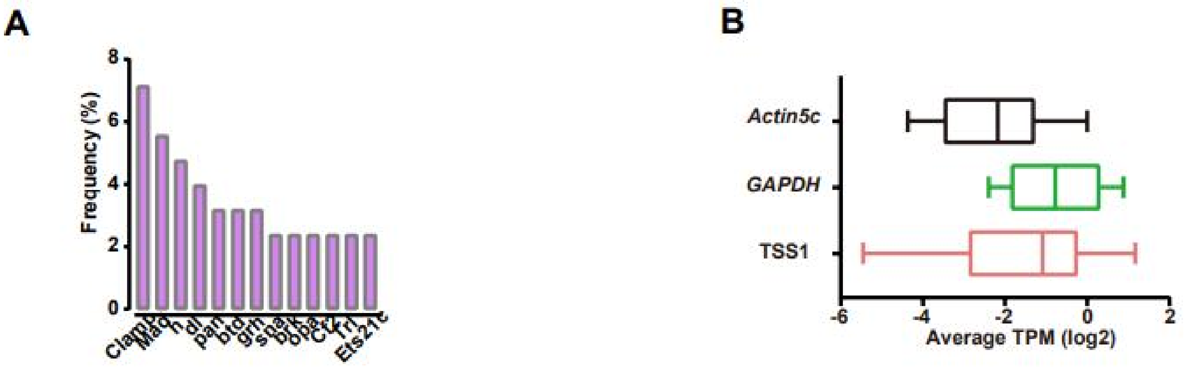

Appendix Figure S3. Potential TFs within the DHS1 and the expression variety of *henna* TSS1.

A Types of TFBSs with top six frequency within the DHS1. The insect core database, which includes GAF binding sites, was downloaded from the JASPAR website, part of the MEME Suite, was then employed to scan genomic sequences for significant matches to TFBS motifs. The open chromatin regions were input as regions of interest. Background letter frequencies in MEME files were changed to the genomic frequencies of the locust genome.

B The variation distribution of gene expression quantity of TSS1, *GAPDH*, and *actin5c*. The expression level was quantified as Log2 TPM.

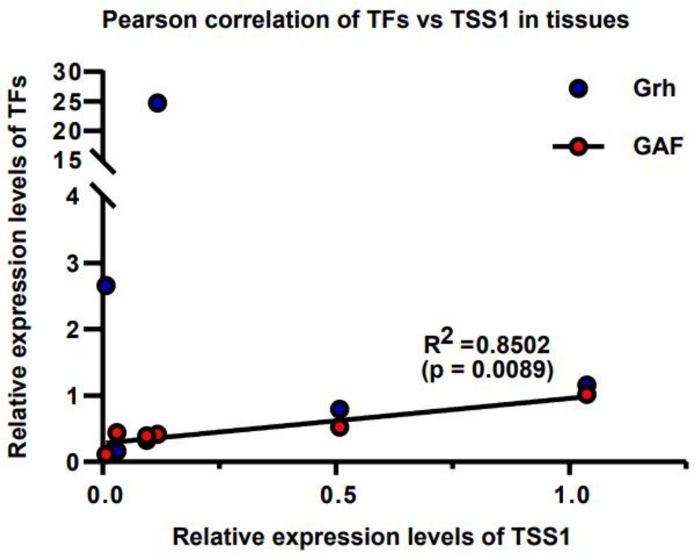

Appendix Figure S4. A positive correlation of GAF versus TSS1 expression in tissues.. $R^2 = 0.8502$ and $P = 0.0089$.

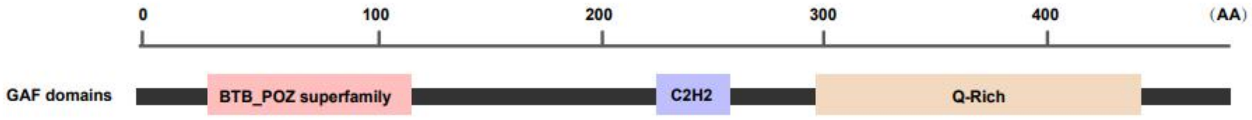

Appendix Figure S5. The protein domains of locust GAF. A schematic of domains of GAF in locusts**.**

A

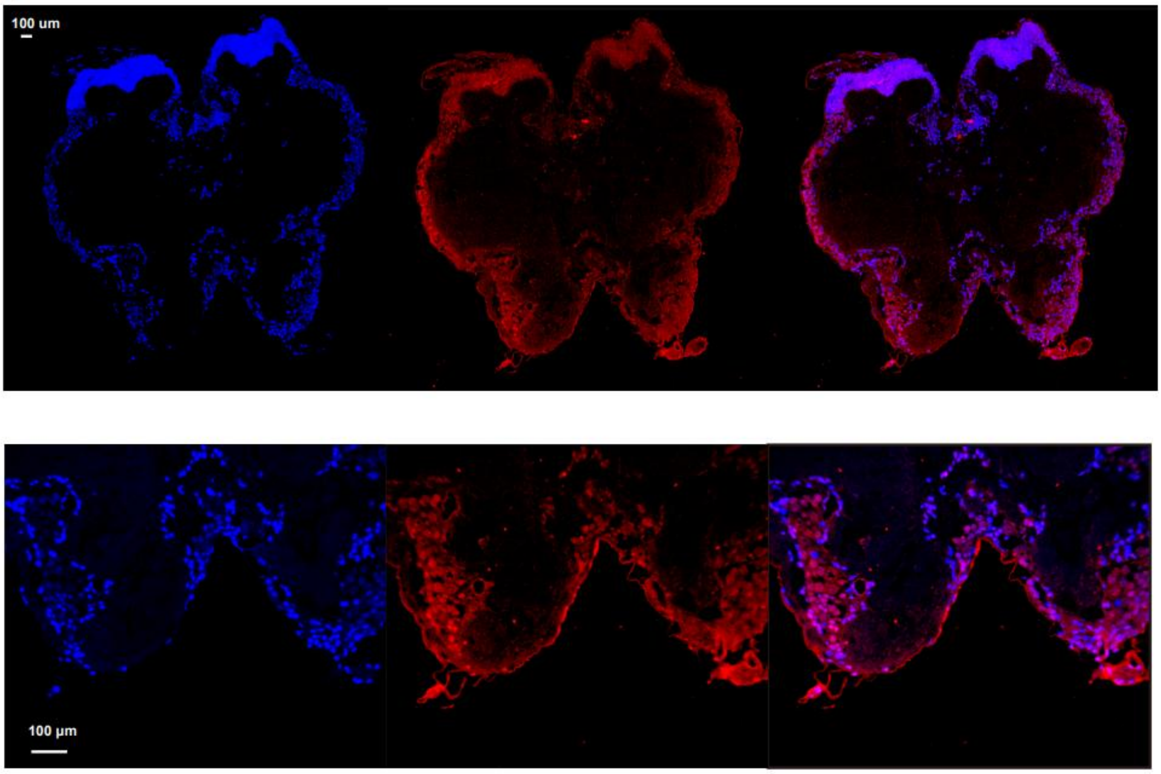

B

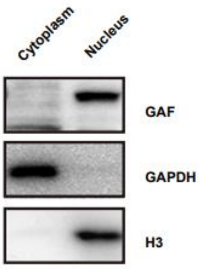

Appendix Figure S6. The protein location of GAF was at nucleus.

A GAF located at nucleus by immunofluorescence of paraffin sectioning. Blue, Hoechst; Red, GAF. Scale bar, 100 μm.

B GAF located at nucleus by Western Blotting. GAPDH is a marker for cytoplasm and H3 is a marker for nucleus.

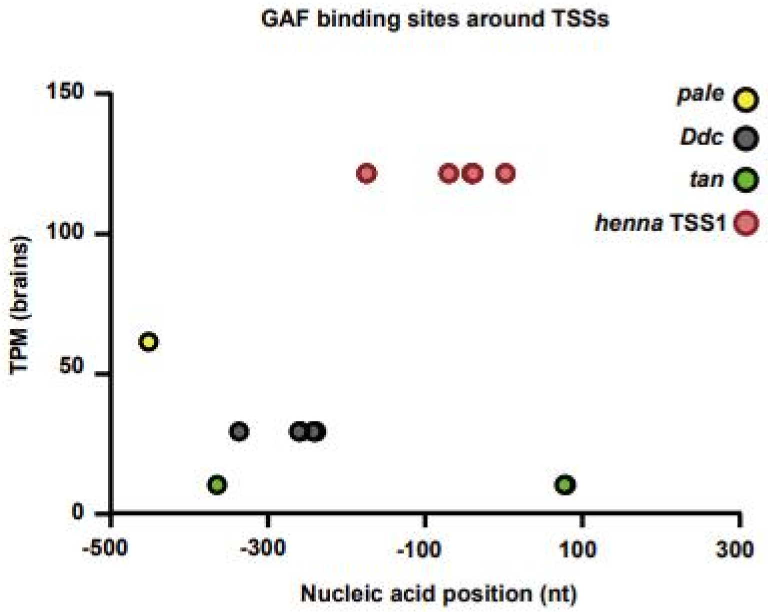

Appendix Figure S7. TPM versus positions of GAF binding sites around *pale*, *Ddc*, *tan*, and *henna* TSS1 in brains.

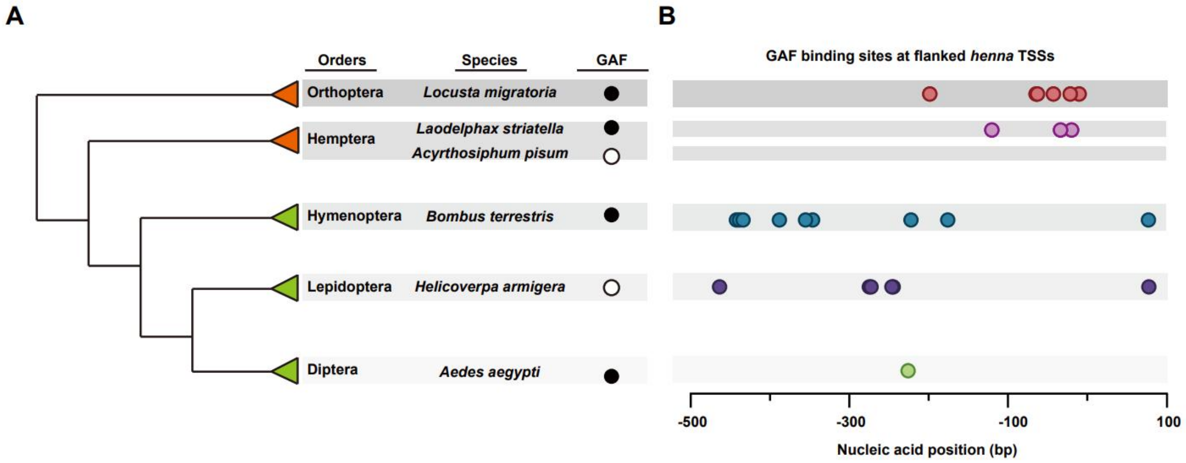

Appendix Figure S8. The widespread presence of multiple GAF binding sites in the flanking region of *henna* TSSs.

A The presence of GAF in several holometabolous (red triangle) and hemimetabolous (green triangle) insects. Black and white circles represent the existence of GAF or not.

B The widespread presence of multiple GAF binding sites in the flanking region of *henna* TSSs.

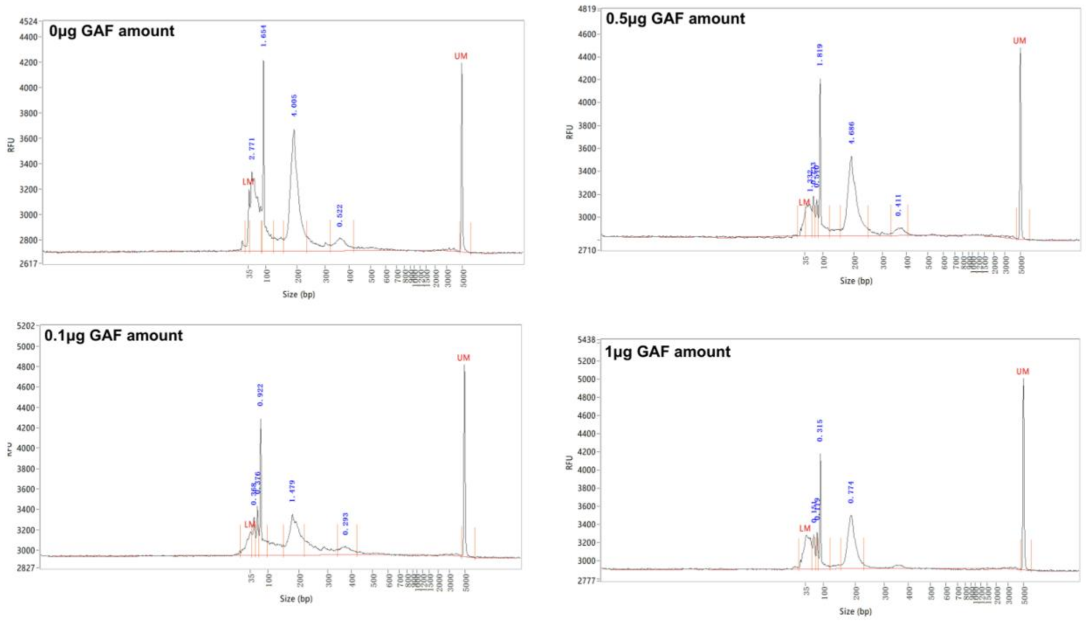

Appendix Figure S9. Example diagrams of DNA fragment quantitative analysis assays using the Agilent 2100 Bioanalyzer system for different GAF amounts, including 0, 0.1, 0.5, 1 µg.

Appendix Table S1. Primers used for DNase-qPCR, ChIP-qPCR, CRISPR/Cas9 PCR, qRT-PCR, and RNAi

Primer names	Primer sequences, 5'-3'	
rp49-DHS-F	ATTTAGTGTATGCTGATTTTGTTTCCA	
rp49-DHS-R	TATTACACTTTATCTTCTTTTCAACTTCTCA	
qhn1-DHS-ReI-F	GACGCATCAGGACCTAAACTT	
qhn1-DHS-ReI-R	GCTGGCGATTCCCAATT	
qhn1_DHS_ReII_F1	TTCCCCTCAGCGGTTTT	
qhn1_DHS_ReII_R1	GCACTACGTCCGTCTTTTG	
qhn1_DHS_ReII_F2	CGATCGGATCTAGTTTACTGGC	
qhn1_DHS_ReII_R2	AGTTATATATATGAGAGCGCGCC	**DNase-qPCR**
qhn1_DHS_ReII&III_F	CCGGCAGTGGAAGAGGG	**ChIP-qPCR**
qhn1_DHS_ReII&III_R	GCTCGCCACCTGCTCTG	
qhn2-DHS-F	GCTGCTCAGCCCGAATG	
qhn2-DHS-R	GTTATTCCGGTCGGTGGC	
qPCR_Inaccessible_F	CGTGTCCATCGCTTTACAGAGG	
qPCR_Inaccessible_R	TGTAGGAATGCCCAGGTGGTTG	
DHS1-mutated-HA-F	AGTGGAAGAGGGGGAGGGGGGCGTGCGTGCAGGCGCGCTCTCATATATATATTTAGTGTATGCTGAT	
DHS1-mutated-HA-R	CCACCTGCTCTGGCCCCGCGCTGTCTACGGCGGCGGCCGCTCACCCAGTTTATTACACTTTATCTTCTTTTCAAC	
Inserted seq-F	tatcGGTACCATTTAGTGTATGCTGAT	**CRISPR/Cas9**
Inserted seq-R	tatcAAGCTTTATTACACTTTATCTTCTTTTCAAC	**PCR**
DHS1-gRNA-F	TAATACGACTCACTATAGCGCGCTCTCATATATATA	
DHS1-gRNA-R	TTCTAGCTCTAAAACGTTATATATATGAGAGCGCG	
Hn-insertest-2-F	CGTAATTGGGAATCGCCAGC	
Hn-insertest-2-R	GTTGCCCGGTGTGTGTAGTC	
qPCR_hn1_F1	GGCAGGCGCAGCGACT	
qPCR_hn1_R1	GTCTTCGACGATCTCTTCGGG	
qPCR_hn2_F	TCTCGCCGGCCGTACA	**qRT-PCR**
qPCR_hn2_R	CATTCGGGCTGAGCAG	

qPCR_hn1_F2	CCCCGGCAGTGGAAGA	
qPCR_hn1_R2	GCTCGCCACCTGCTCTG	
Hn-exon2F	GATGCTGATGACGGGAGGC	
Hn-exon2R	CTTGAGGCATTTGGCGAGT	
qPCR_Grh_F	AGGACGACCGCATTAGC	
qPCR_Grh_R	AACTGCCACGCCTTGAT	
qPCR_GAF_F1	TCTGCGGTTCAACTCCTG	
qPCR_GAF_R1	GCAAAGACGGCAACTGATT	
qPCR_GAF_F2	GTTGCACCAAGTAGTTCAAAT	
qPCR_GAF_R2	CTTTAGAAGGTCCAAAAGAAA	
RNAi_hn1_F	GAAGCGGGCAGACGAGA	
RNAi_hn1_R2	GTCTTCGACGATCTCTTCGG	
T7-RNAi_hn1_F	TAATACGACTCACTATAGGGAAGCGGGCAGACGAGA	
T7_RNAi_hn1_R2	TAATACGACTCACTATAGGGTCTTCGACGATCTCTTCGG	
RNAi_hn2_F	TTCGTGAGGCGGCACTC	
RNAi_hn2_R	GGCGAGAAGATGAGGCAAAT	
T7-RNAi_hn2_F	TAATACGACTCACTATAGGTTCGTGAGGCGGCACTC	**RNAi**
T7_RNAi_hn2_R	TAATACGACTCACTATAGGGGCGAGAAGATGAGGCAAAT	
dsGAF_F	AGGGTCTGGTGCCTCAAA	
dsGAF_R	CCGACATGCGGTACTTACA	
T7_dsGAF_F	TAATACGACTCACTATAGGAGGGTCTGGTGCCTCAAA	
T7_dsGAF_R	TAATACGACTCACTATAGGCCGACATGCGGTACTTACA	

Appendix Table S2. Primers used for recombinant plasmid construction

Primer names	Primer sequences, 5'-3'	
HindIII_hn1_TSS_F	AAATAAAGCTTGGCGGCCGCTCACCCAGTTAT	
XhoI_hn1_5end_R	AAATACTCGAGTCAGGACCTAAACTTTCCTGT	
HindIII_hn2_TSS_F	AAATAAAGCTTTGGGGCCTTTGGGTGG	**Promoter reporter assay**
HindIII_hn2_DHS_F	AAATAAAGCTTGAGGAGAGTTTCCTTCGTGAGG	
HindIII_hn2_ATG_F	AAATAAAGCTTCATGTCTGGGCGTCGT	
XhoI_hn2_5end_	AAATACTCGAGGTTATTCCGGTC	

R	GGTG	
KpnI_Re_GAF_F	AAATAGGTACCCTGCCGGCTCTG<br>CTGTGCTAC	
BstBI_Re_GAF_<br>R	AAATATTCGAAATGGGGAGCAG<br>TCAGTTGTACAGT	**Recombinant expression vector**
KpnI_GAF_F	AATAGGTACCATGGGGAGCAGT<br>CAGTTGTACAGTT	
BstBI_GAF_R	ATATTCGAAA<br>CTGCCGGCTCTGCTGTGCTA	
