# Supplementary figures and images for: GAF-dependent chromatin plasticity determines promoter usage to mediate locust gregarious behavior
Source: EMBO J. 2025 Apr 7;44(10):2928–48. doi: 10.1038/s44318-025-00428-x (PMC12084303; doi:10.1038/s44318-025-00428-x)

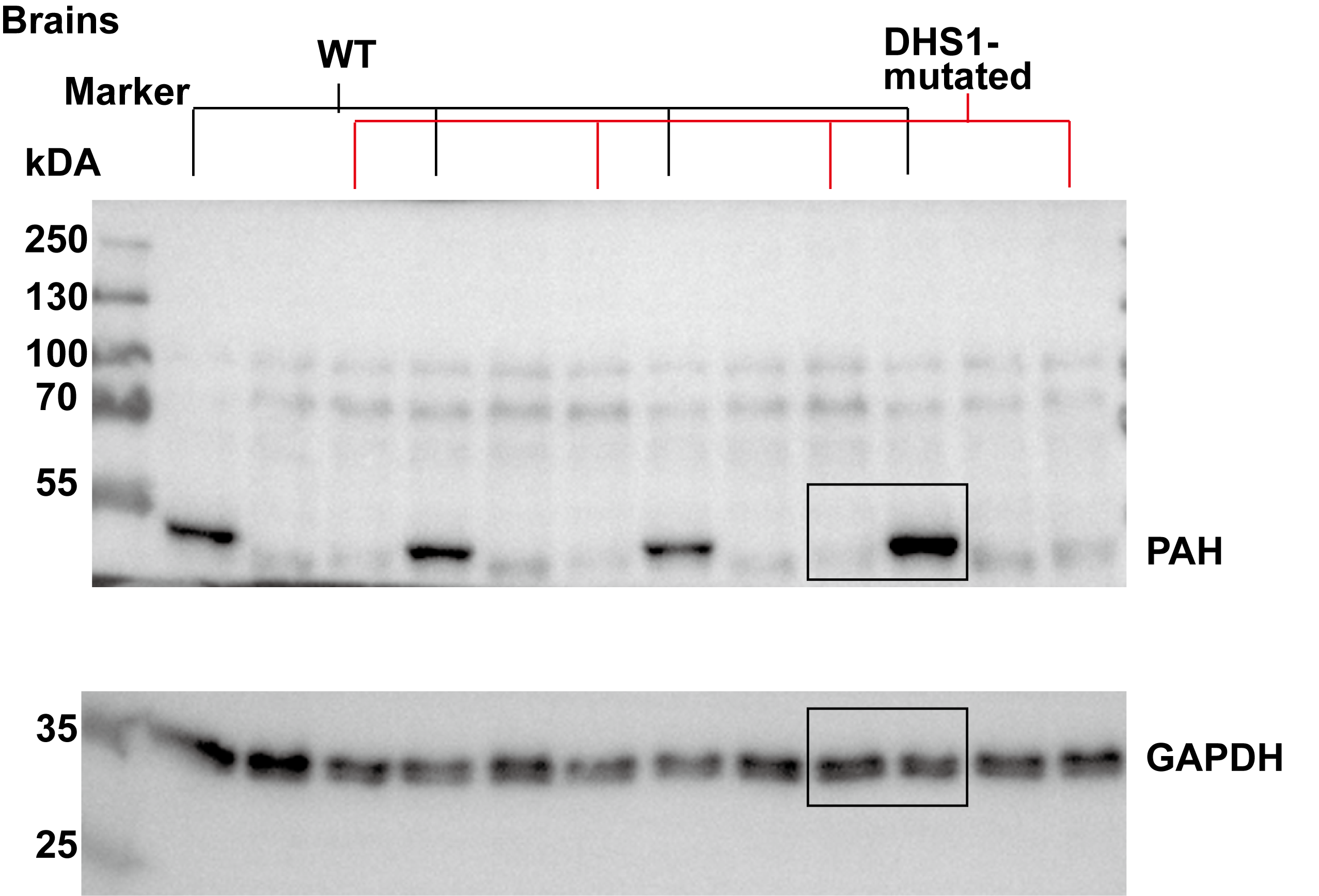

Supplement: Supplementary file 3 — Source data Fig. 1 [file 44318_2025_428_MOESM3_ESM.zip › Figure 1/1E/Western Blot-Brains-PAH&GAPDH.tif]

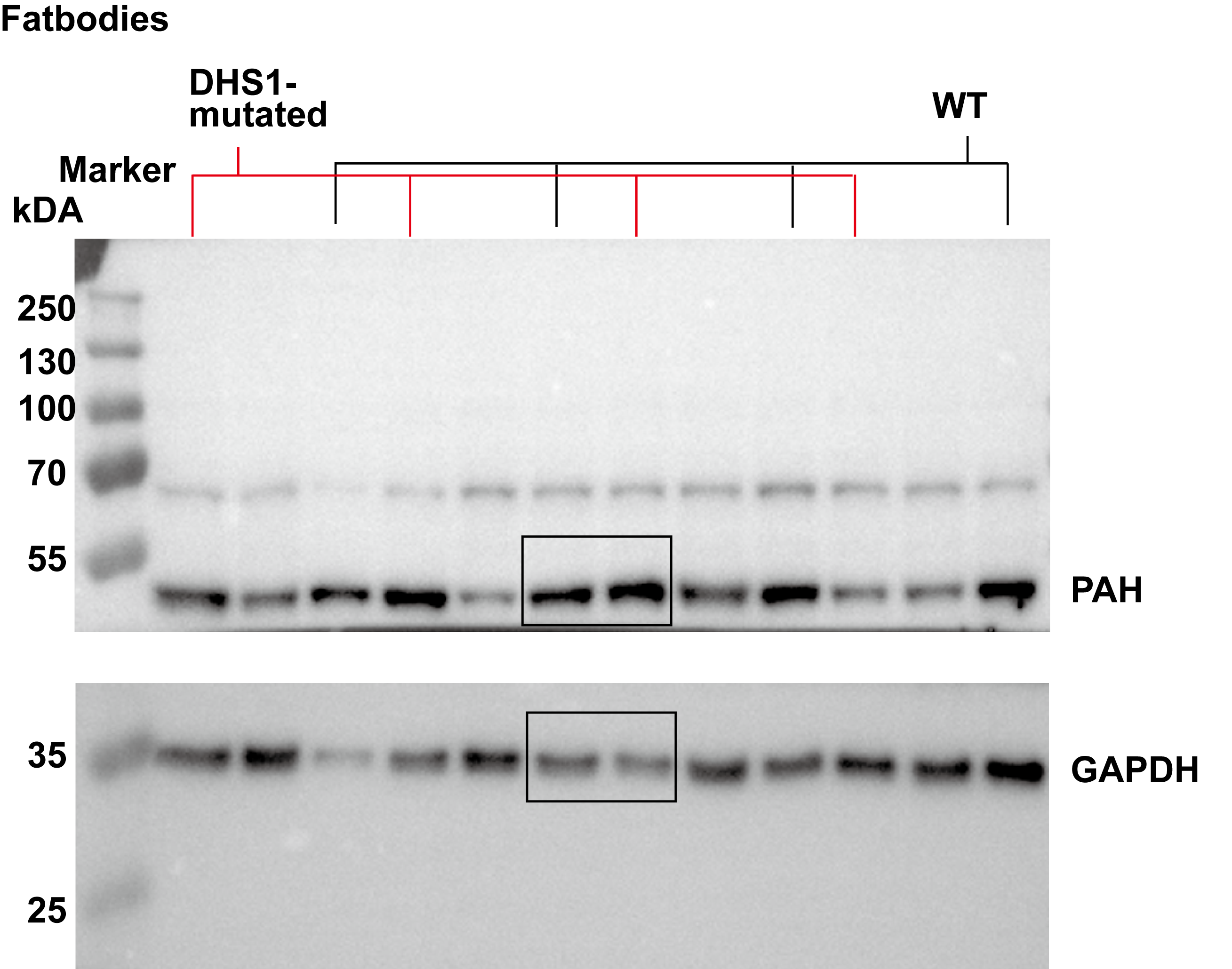

Supplement: Supplementary file 3 — Source data Fig. 1 [file 44318_2025_428_MOESM3_ESM.zip › Figure 1/1E/Western Blot-Fatbodies-PAH&GAPDH.tif]

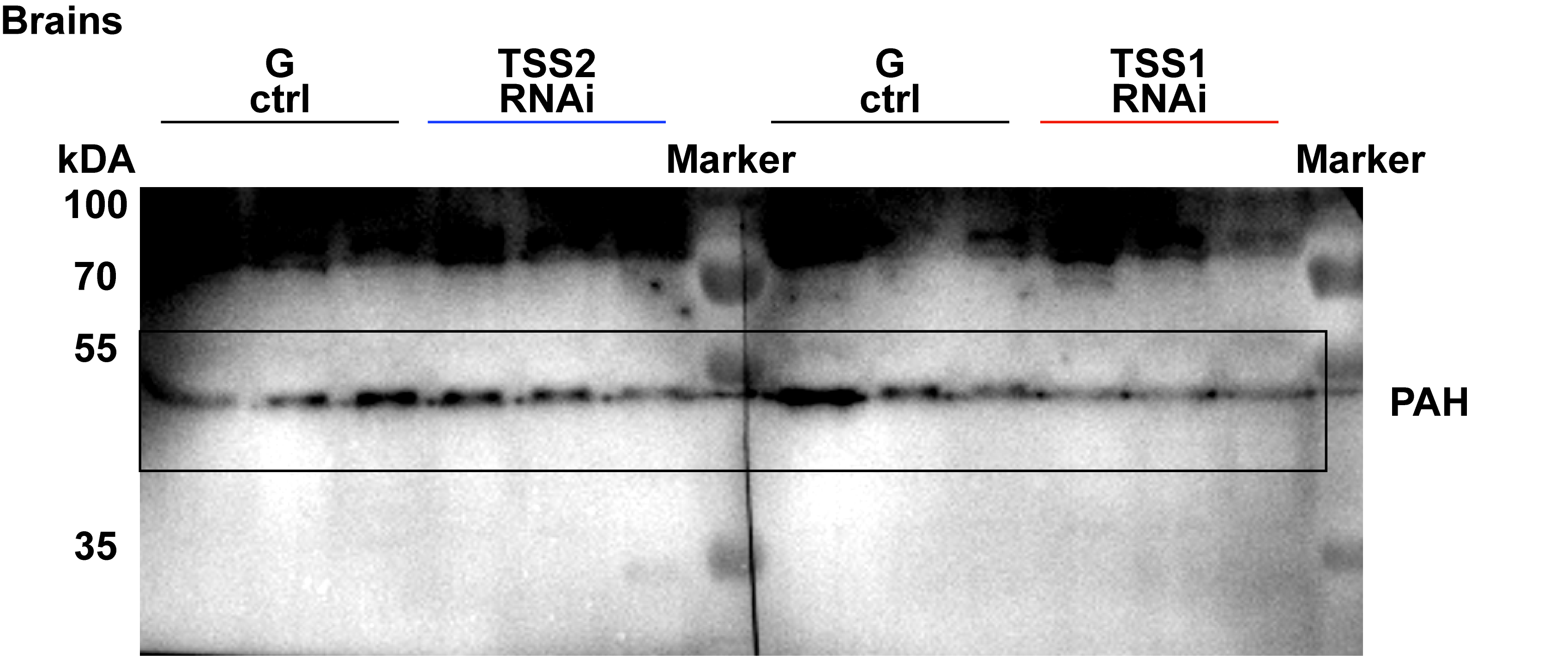

Supplement: Supplementary file 5 — Source data Fig. 3 [file 44318_2025_428_MOESM5_ESM.zip › Figure 3/3D/Western Blot-PAH.tif]

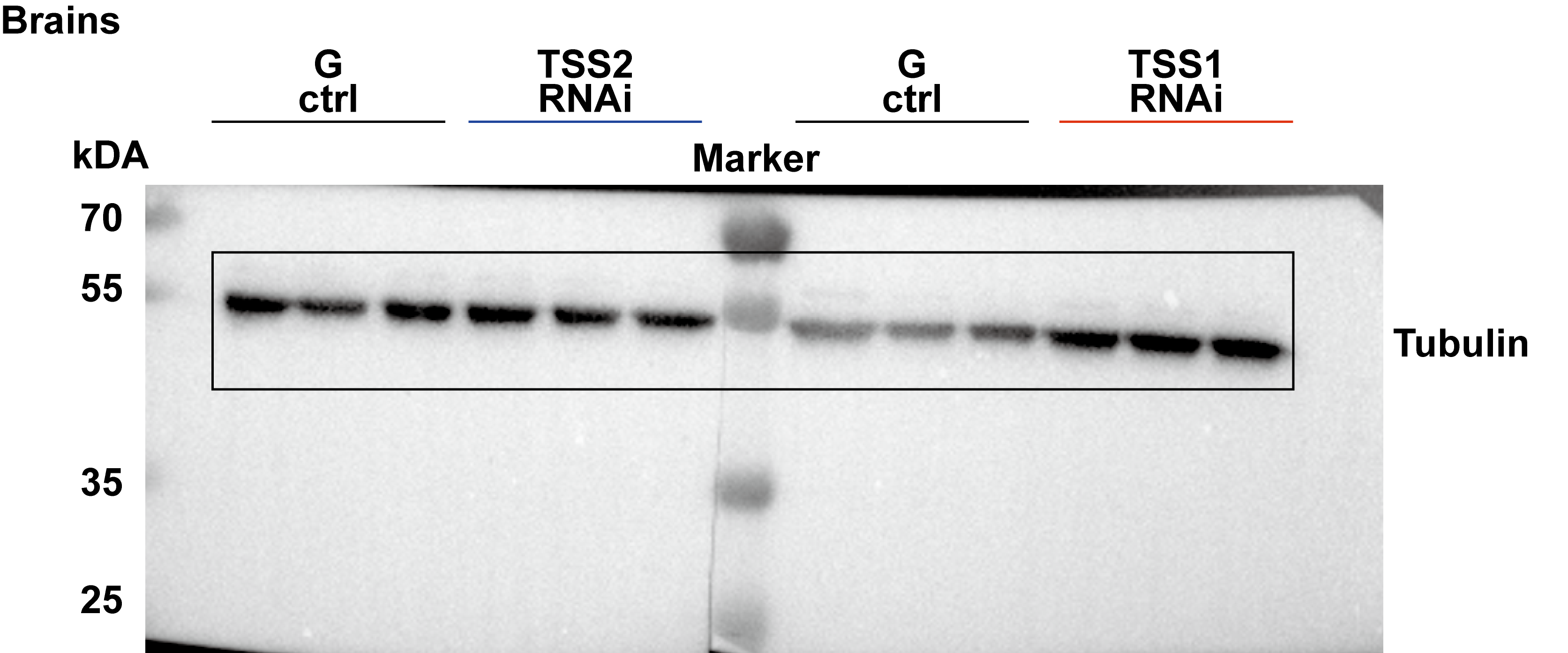

Supplement: Supplementary file 5 — Source data Fig. 3 [file 44318_2025_428_MOESM5_ESM.zip › Figure 3/3D/Western Blot-Tubulin.tif]

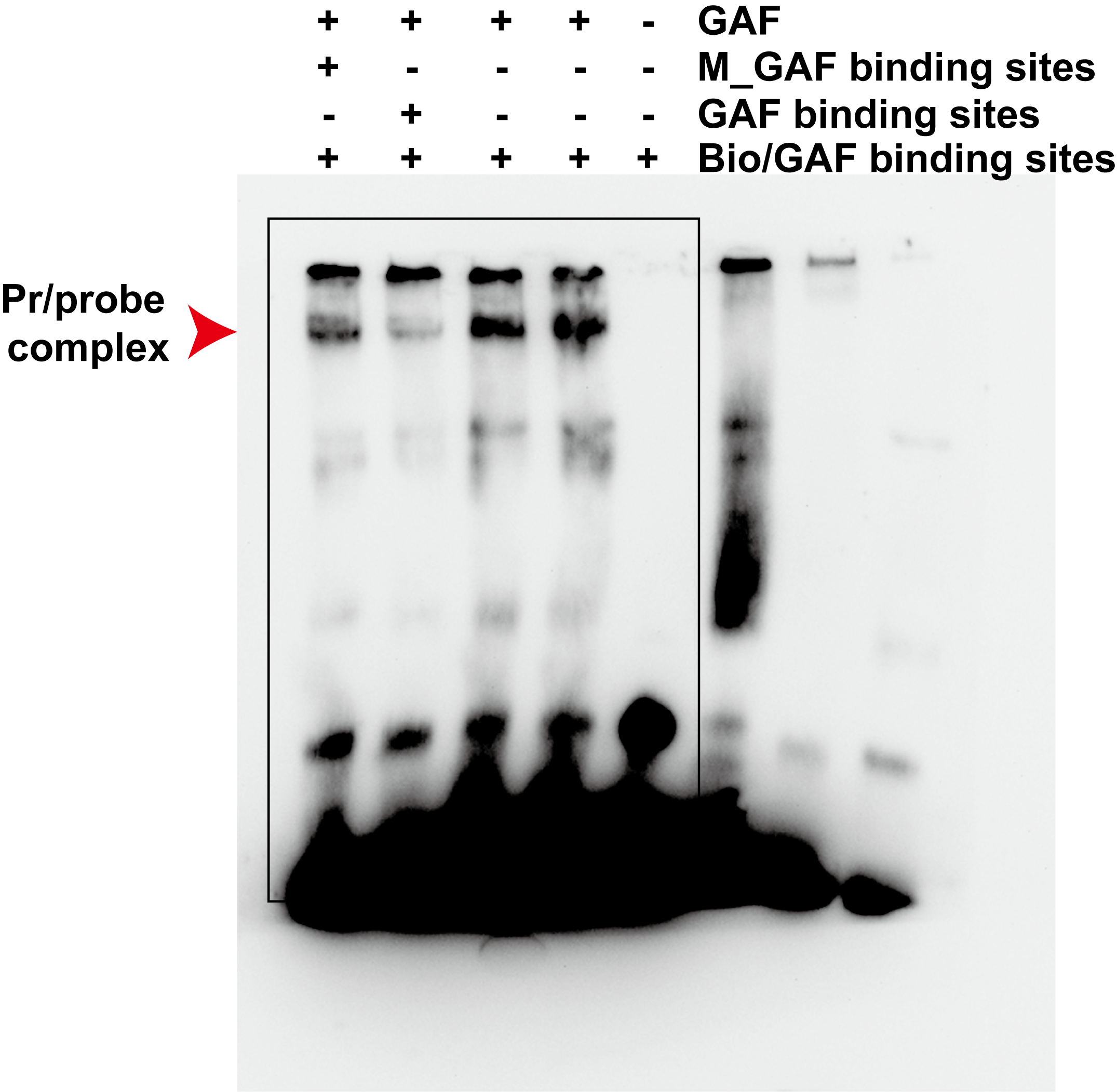

Supplement: Supplementary file 6 — Source data Fig. 4 [file 44318_2025_428_MOESM6_ESM.zip › Figure 4/4H/EMSA.tif]

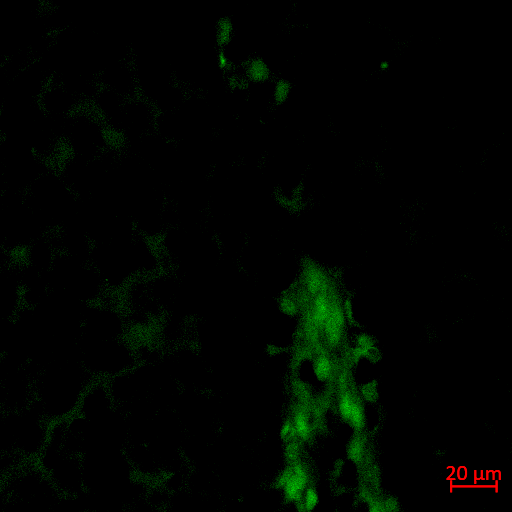

Supplement: Supplementary file 6 — Source data Fig. 4 [file 44318_2025_428_MOESM6_ESM.zip › Figure 4/4M/IF_Fr-GAF_FITC-63X-Ch2.tif]

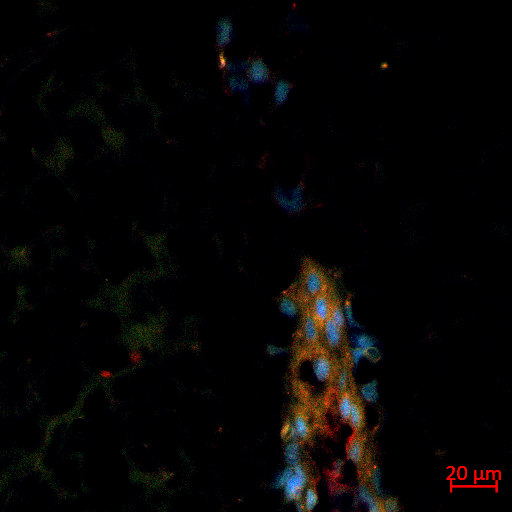

Supplement: Supplementary file 6 — Source data Fig. 4 [file 44318_2025_428_MOESM6_ESM.zip › Figure 4/4M/IF_Fr-Merge-63X.tif]

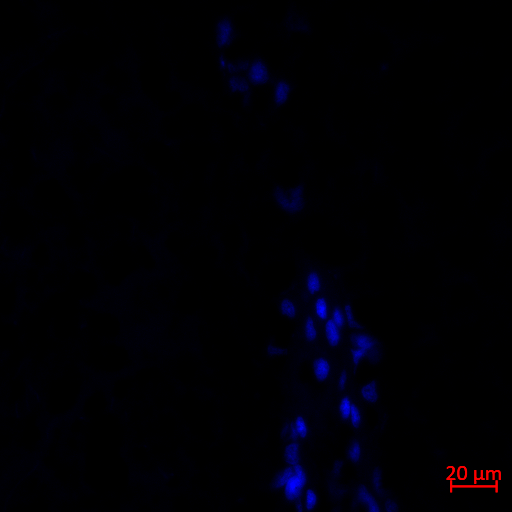

Supplement: Supplementary file 6 — Source data Fig. 4 [file 44318_2025_428_MOESM6_ESM.zip › Figure 4/4M/IF_Fr-Nuclei-63X-Ch1.tif]

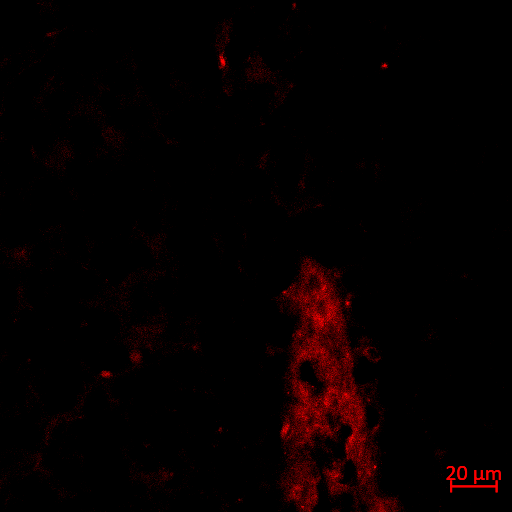

Supplement: Supplementary file 6 — Source data Fig. 4 [file 44318_2025_428_MOESM6_ESM.zip › Figure 4/4M/IF_Fr-PAH_alex546-63X-Ch3.tif]

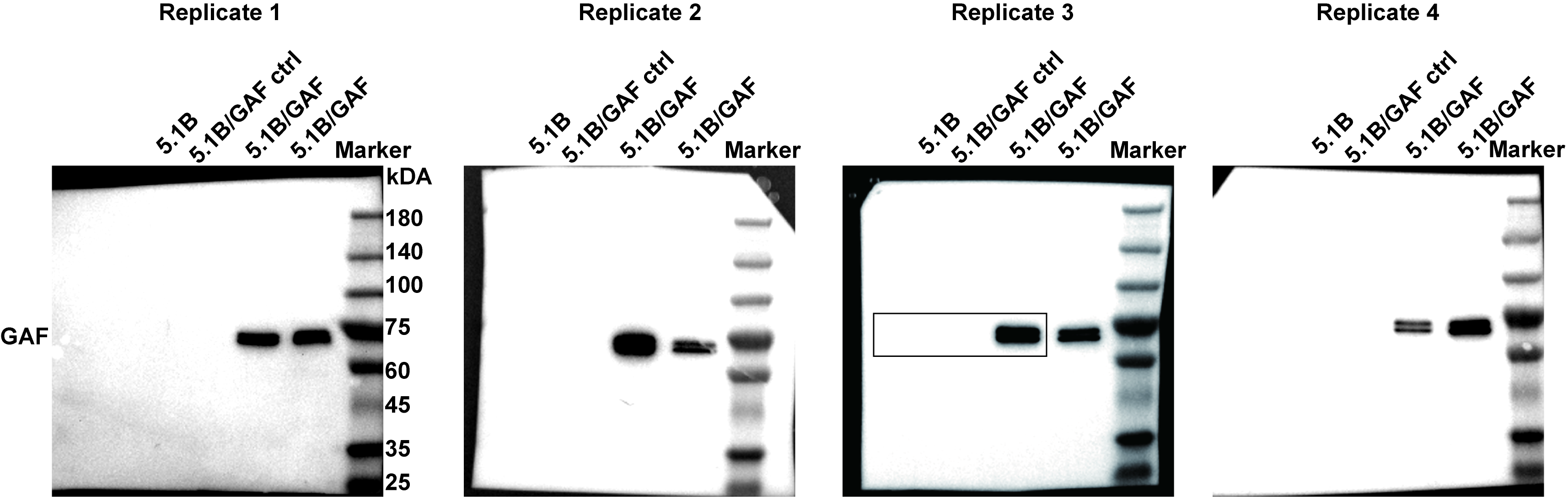

Supplement: Supplementary file 7 — Source data Fig. 5 [file 44318_2025_428_MOESM7_ESM.zip › Figure 5/5B/Western blot-GAF.tif]

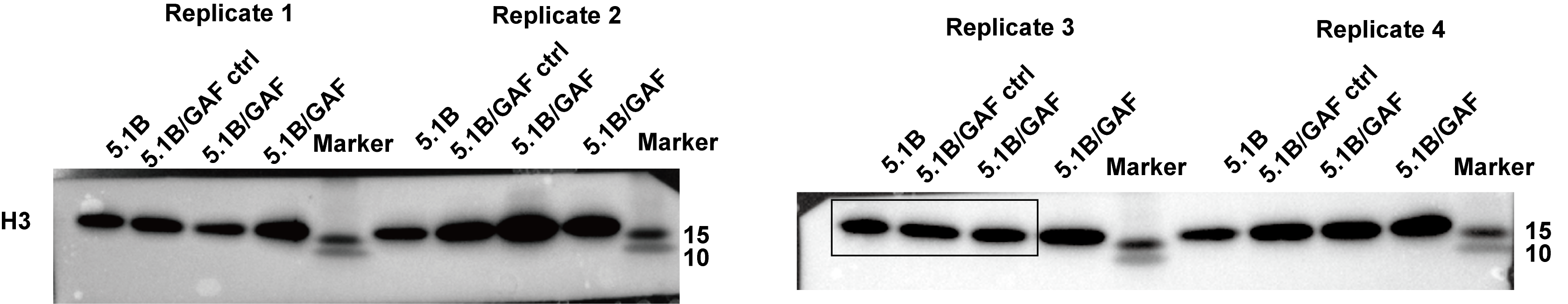

Supplement: Supplementary file 7 — Source data Fig. 5 [file 44318_2025_428_MOESM7_ESM.zip › Figure 5/5B/Western blot-H3.tif]

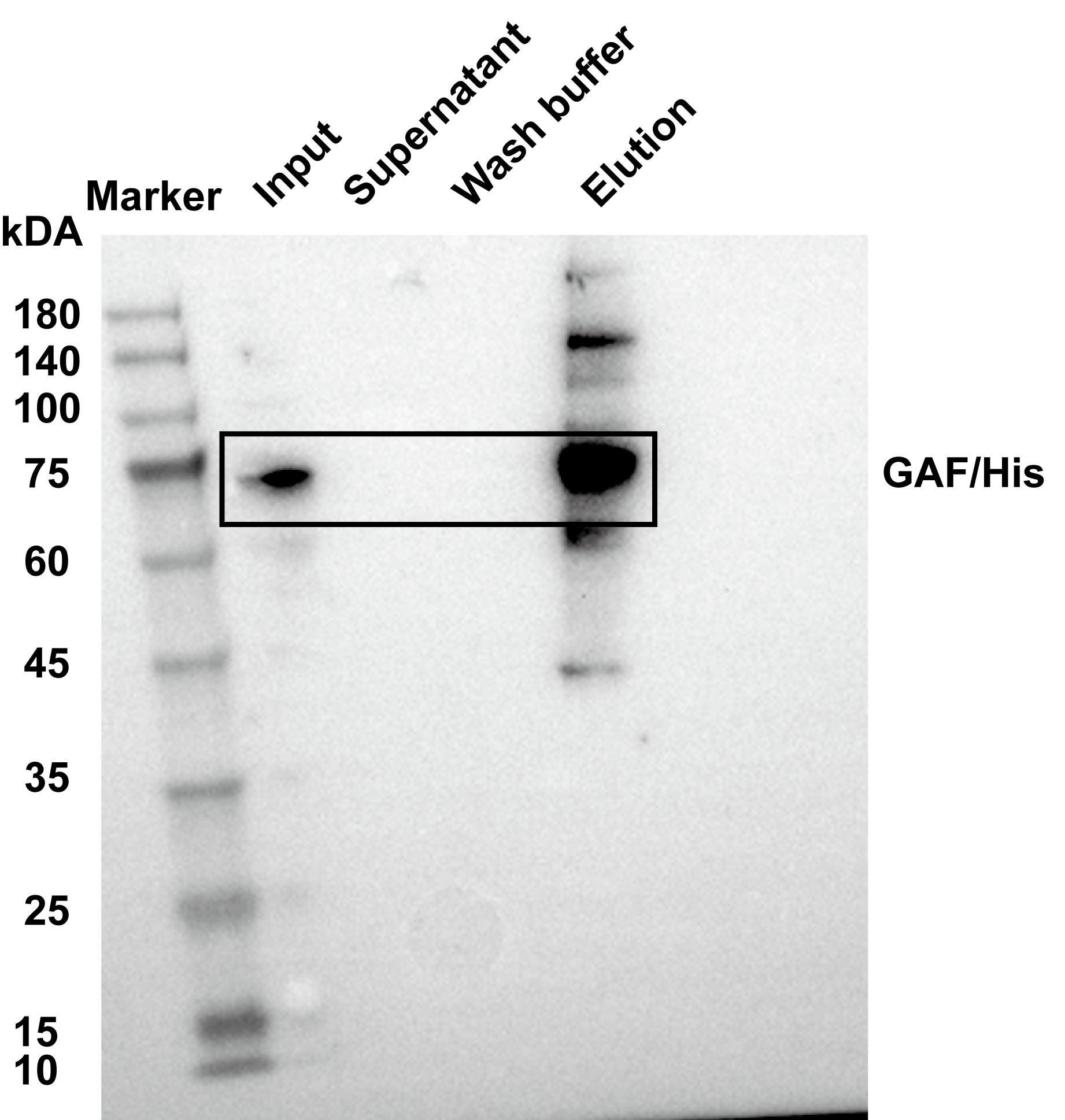

Supplement: Supplementary file 8 — Source data Fig. 6 [file 44318_2025_428_MOESM8_ESM.zip › Figure 6/6E/Western Blot-GAF.tif]

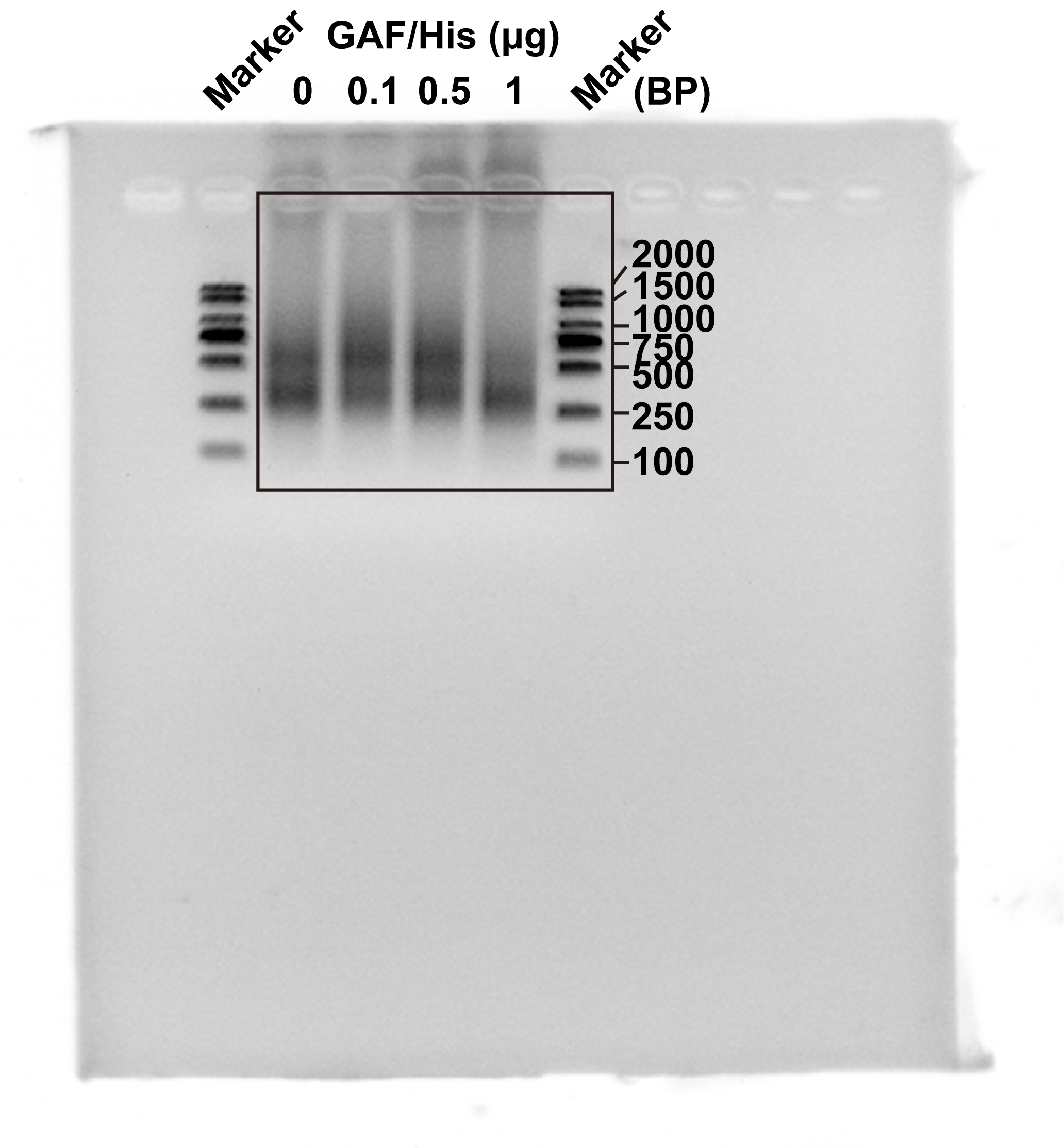

Supplement: Supplementary file 8 — Source data Fig. 6 [file 44318_2025_428_MOESM8_ESM.zip › Figure 6/6F/Agarose Gel.tif]

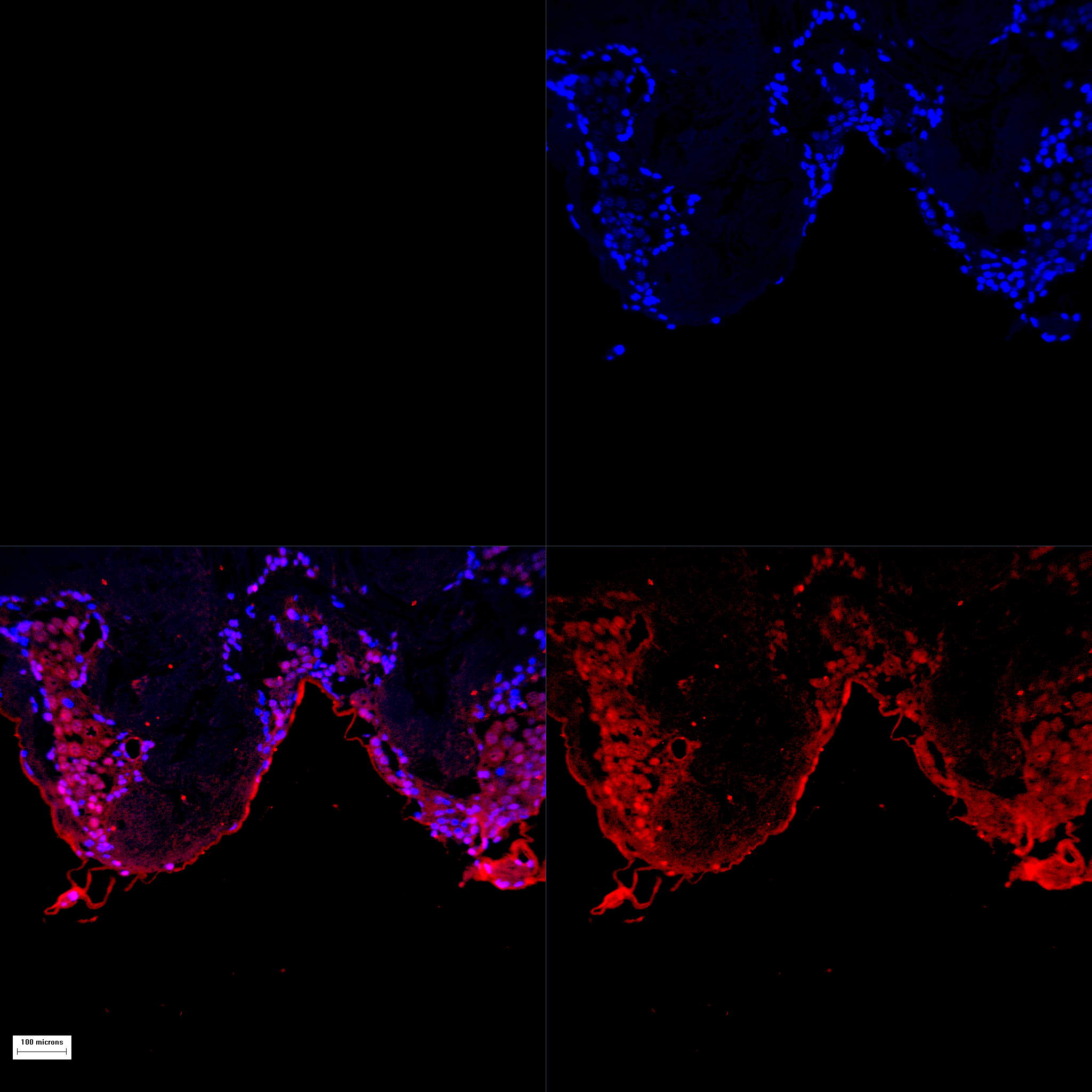

Supplement: Supplementary file 9 — EV and Appendix Figure Source Data [file 44318_2025_428_MOESM9_ESM.zip › Appendix/Appendix Figure S6/6B/IF-para-20X.tif]

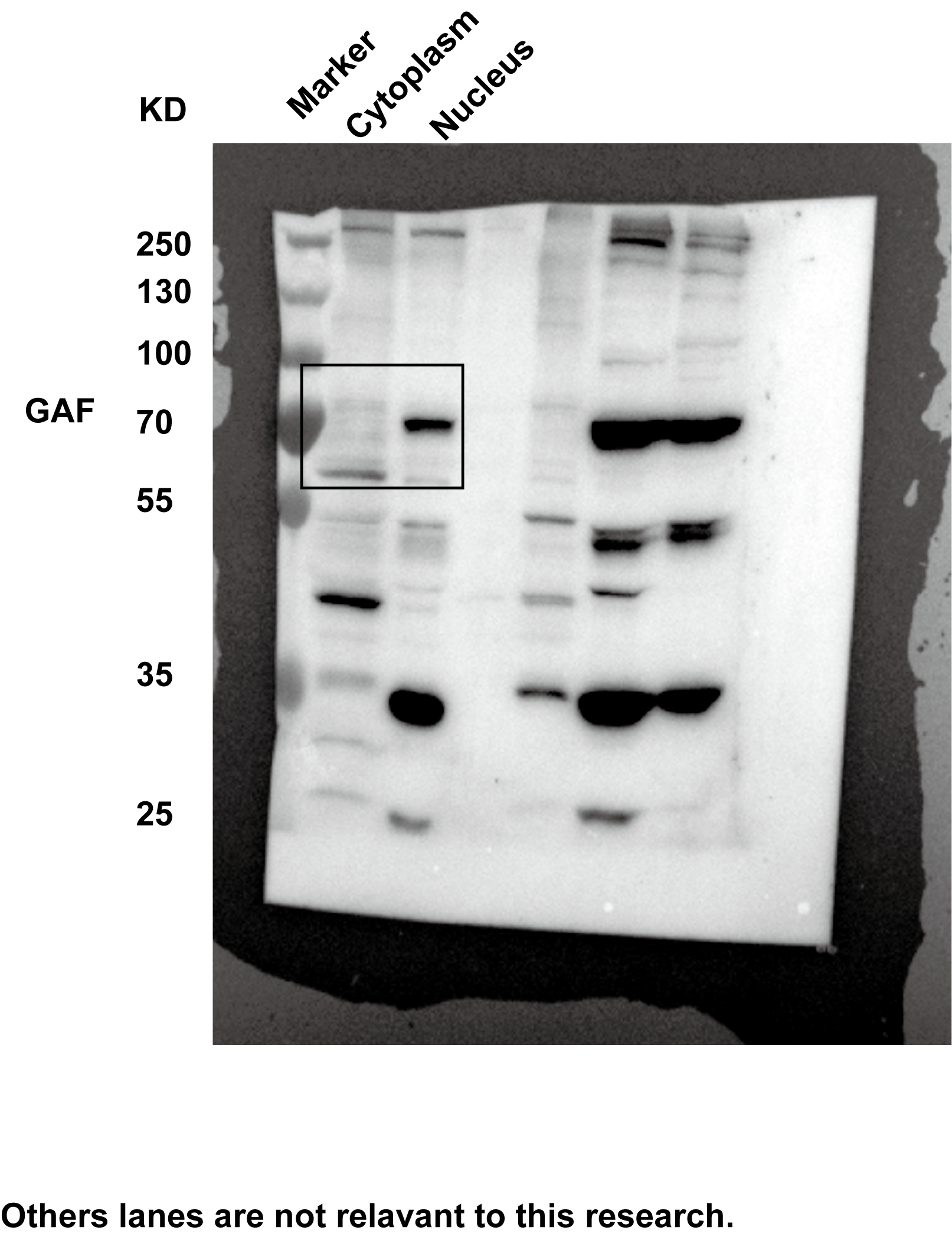

Supplement: Supplementary file 9 — EV and Appendix Figure Source Data [file 44318_2025_428_MOESM9_ESM.zip › Appendix/Appendix Figure S6/6B/Western Blot-GAF.tif]

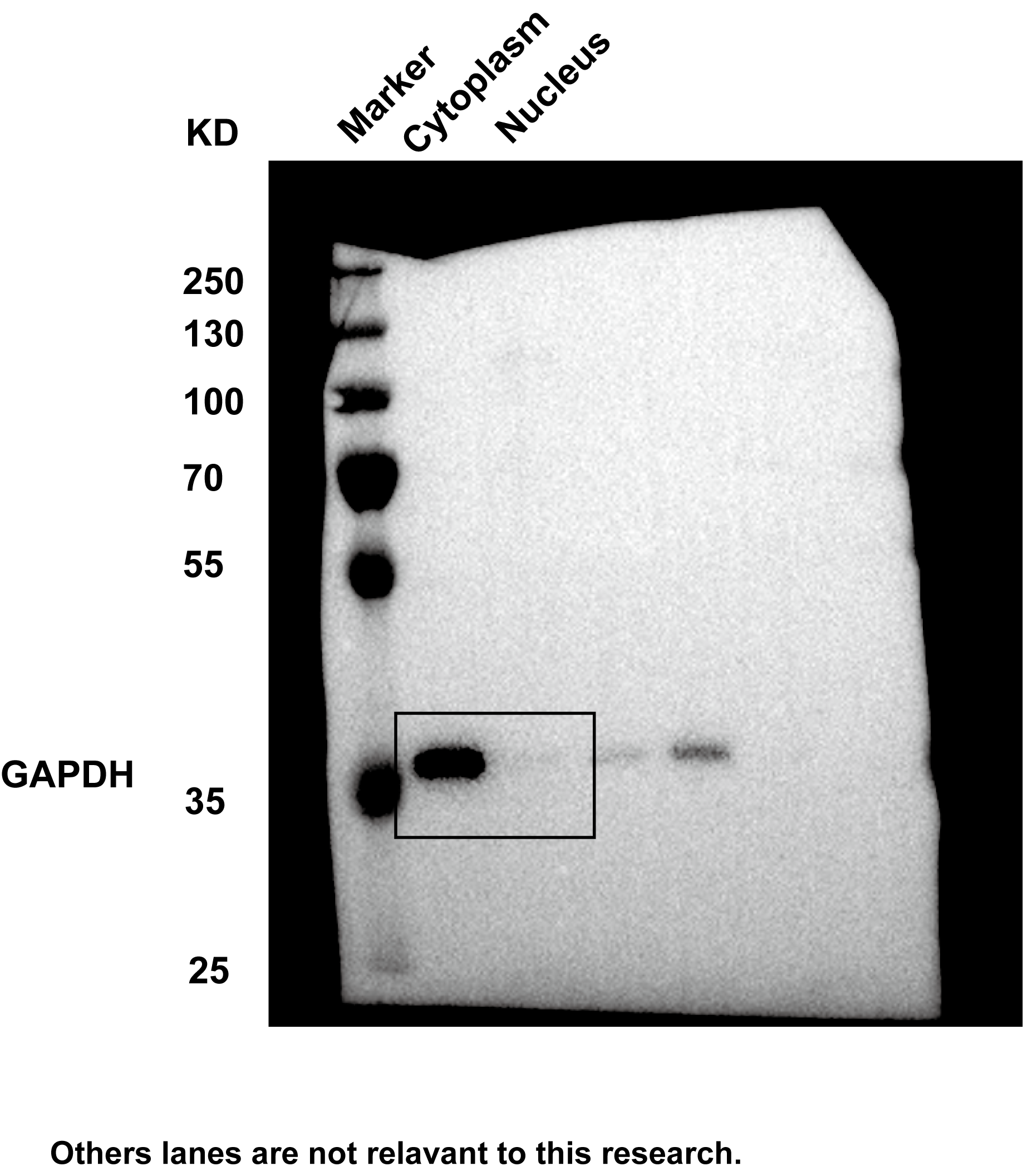

Supplement: Supplementary file 9 — EV and Appendix Figure Source Data [file 44318_2025_428_MOESM9_ESM.zip › Appendix/Appendix Figure S6/6B/Western Blot-GAPDH.tif]

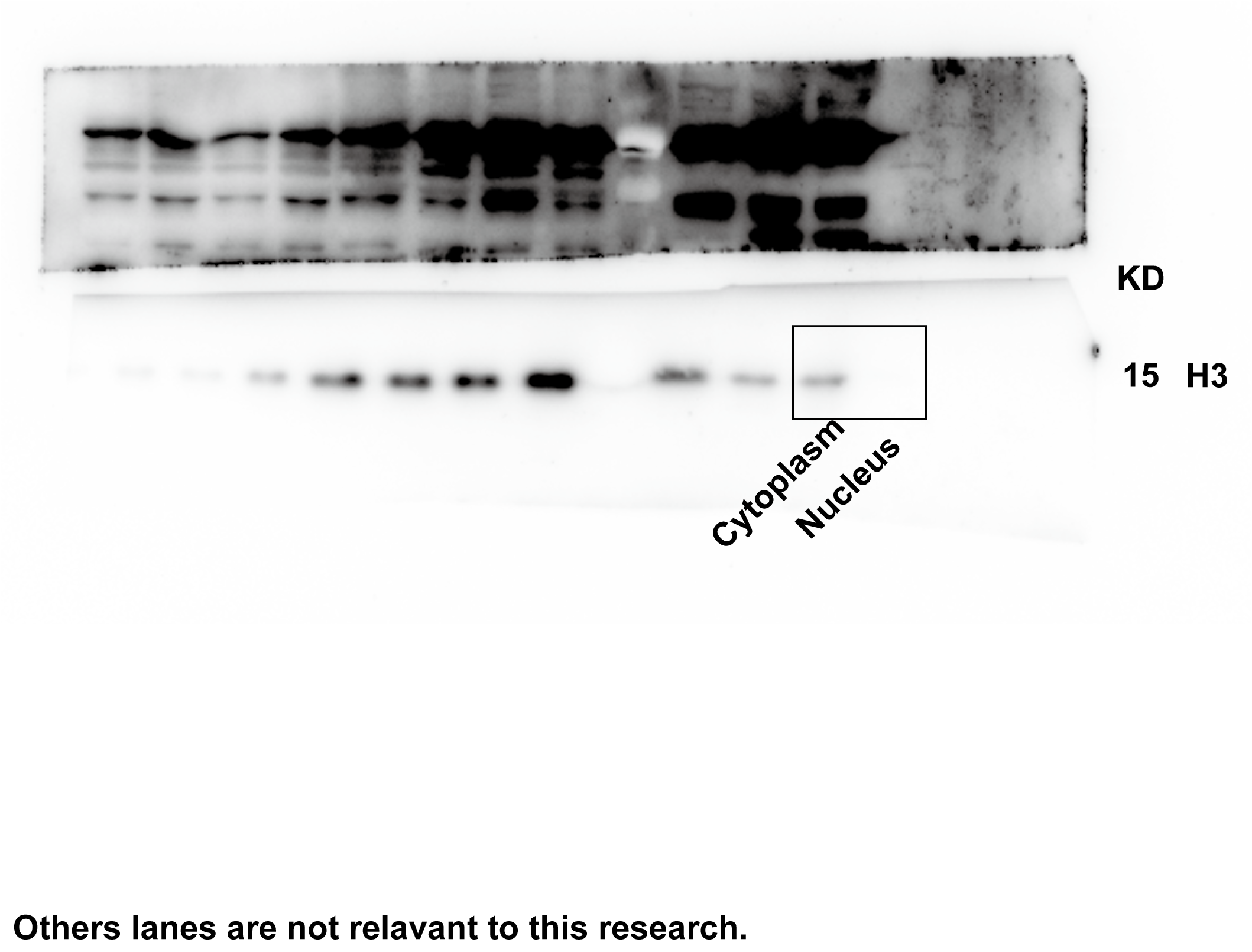

Supplement: Supplementary file 9 — EV and Appendix Figure Source Data [file 44318_2025_428_MOESM9_ESM.zip › Appendix/Appendix Figure S6/6B/Western Blot-H3.tif]
