# Supplementary material for: GAF-dependent chromatin plasticity determines promoter usage to mediate locust gregarious behavior
Source: EMBO J. 2025 Apr 7;44(10):2928–48. doi: 10.1038/s44318-025-00428-x (PMC12084303; doi:10.1038/s44318-025-00428-x)
Supplement: Supplementary file 10 — Expanded View Figures [file 44318_2025_428_MOESM10_ESM.pdf]

Expanded View Figures

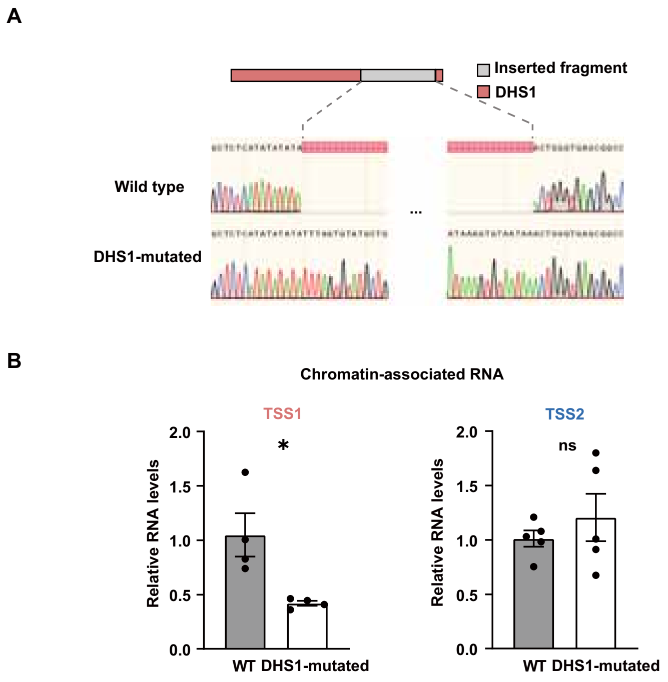

Figure EV1. The chromatin-associated RNA level of TSS1 and TSS2 in the brains of homozygous DHS1-dysfunctional mutants of gregarious locusts.

(**A**) A diagram of the gene structure of homozygous DHS1-dysfunctional mutants of gregarious locusts. (**B**) The chromatin-associated RNA level of TSS1 and TSS2 in the brains of homozygous DHS1-dysfunctional mutants of gregarious locusts. Primers were designed specifically for the TSS. N = 4 for TSS1, and n = 5 for TSS2. WT, wild type. *P* values from left to right, $P = 0.0286$ (Mann–Whitney test), $P = 0.4221$. Values are means and error bars indicate SEM. **P* < 0.05, Student's t tests. Source data are available online for this figure.

 © The Author(s)

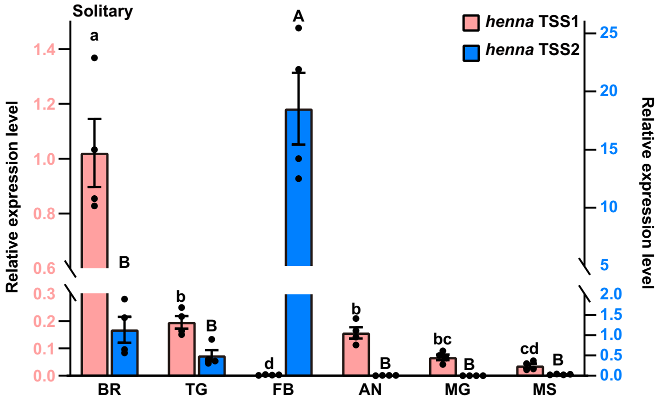

Figure EV2. The mRNA expression pattern for henna TSS1 and TSS2 in solitary locusts.

$N = 4$ for each group. Values are means and error bars indicate SEM. $P = 0.0020$ for TSS1 (Brown–Forsythe ANOVA test), $P < 0.0001$ for TSS2 (ordinary one-way ANOVA tests). BR, Brain. TG, thoracic ganglia. FB, fat body. AN, antenna. MG, Midgut. MS, muscle. Same letters above the bars indicate no significant difference between groups. Different letters indicate a significant difference between groups. Combined letters indicate groups are different from others but not from each other. Source data are available online for this figure.

© The Author(s)

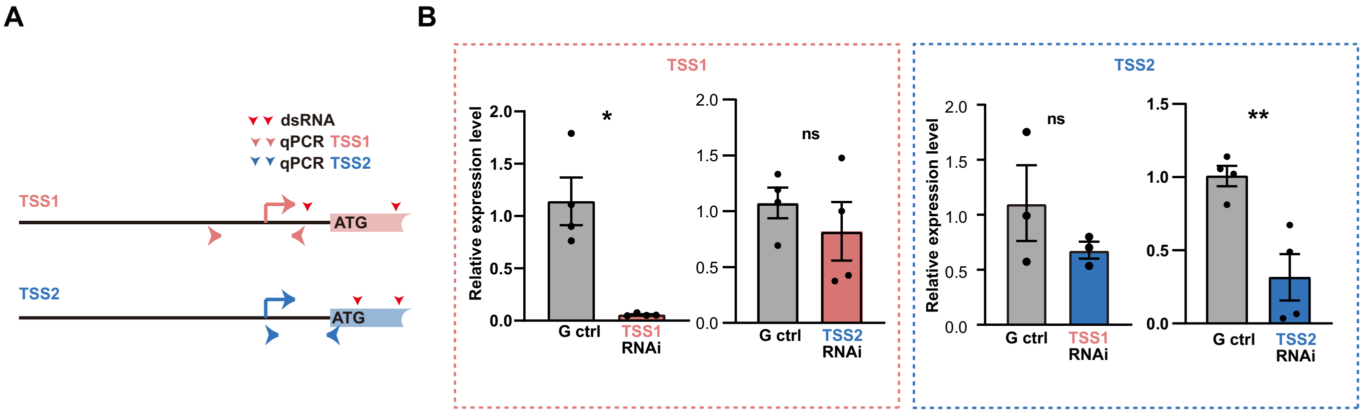

Figure EV3. The reduction efficiency of the henna TSS1 and TSS2 was independent.

(**A**) A schematic diagram of the location of primers for RNAi and qPCR. (**B**) In red dotted box, it was the expression of TSS1 for gregarious control (G ctrl), TSS1 RNAi and TSS2 RNAi (n = 4 for TSS1 groups, G ctrl and TSS2 RNAi of TSS2 groups; $n = 3$ for G ctrl and TSS1 RNAi of TSS2 groups). Of TSS1, P values from left to right, $P = 0.0286$, $P = 0.4213$. Of TSS2, P values from left to right, $P = 0.2932$, $P = 0.0071$. Values are means and error bars indicate SEM. *$P < 0.05$, **$P < 0.01$. Of TSS1, G ctrl versus TSS2 RNAi, and groups of TSS2, Student's t tests. Of TSS1, G ctrl versus TSS1 RNAi, Mann–Whitney test. Source data are available online for this figure.

© The Author(s)

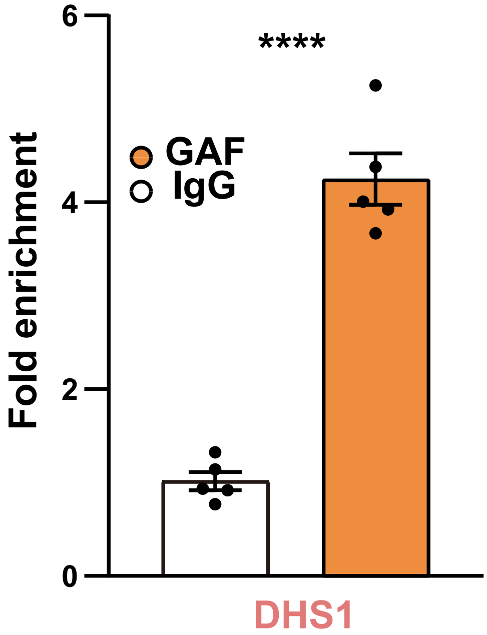

Figure EV4. The fold enrichment of GAF binding at DHS1 in vivo.

$N = 5$ for each group. $P = 4.02e\text{-}6$. Values are means and error bars indicate SEM. ****$P < 0.0001$, Student's t tests. Source data are available online for this figure.

© The Author(s)
